# P-615. Knowledge, attitudes and practice towards routine vaccination among medical students, Kazakhstan, 2023

**DOI:** 10.1093/ofid/ofae631.813

**Published:** 2025-01-29

**Authors:** Arystan Balmagambetov, Balaussa Zhuman, Roberta Horth, Dilyara Nabirova

**Affiliations:** EOC National Center of Public Health of the MOH KZ, Astana, Astana, Kazakhstan; Scientific and Practical Center for Sanitary and Epidemiological Expertise and Monitoring, Almaty, Kazakhstan, Almaty, Almaty, Kazakhstan; US Centers for Disease Control and Prevention, Dulles, Virginia; CDC Central Asia office, Almaty, Almaty, Kazakhstan

## Abstract

**Background:**

In Kazakhstan, coverage for childhood immunizations has decreased in recent years. Healthcare workers have the ability to increase vaccine coverage if they are sufficiently trained and have correct knowledge, attitudes and practices (KAP) related to routine immunizations. To assess whether interventions are needed in pre-service medical training, we needed to understand current vaccine KAP in medical students.

**Table 1.**
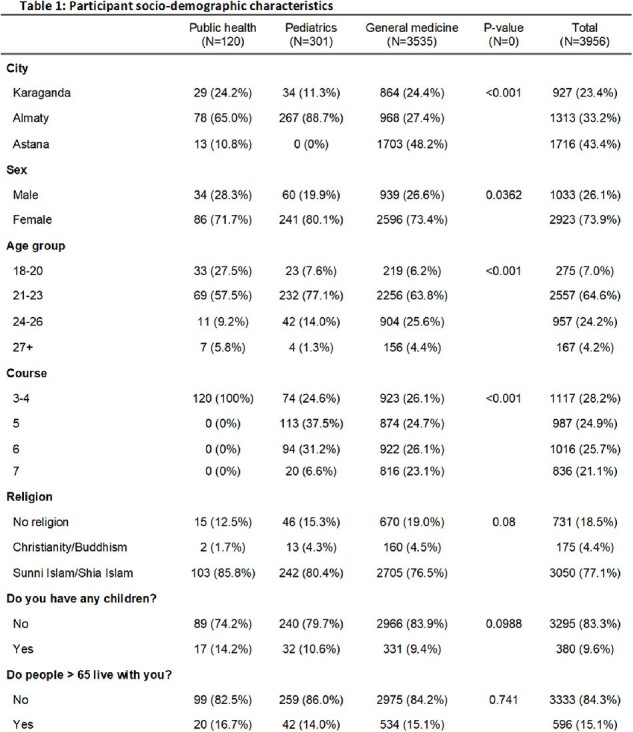
Participant characteristics

**Methods:**

We conducted a cross-sectional study among senior medical students in 3 universities in Karaganda, Astana and Almaty, from March to May 2023. We used multivariable logistic regression to test association with adequate knowledge, attitudes, and practices (KAP); threshold for adequate scores for questions in each domain was 70%. Adjusted odds ratios (OR) and 95% confidence intervals (CI) are reported.

**Figure 1.**
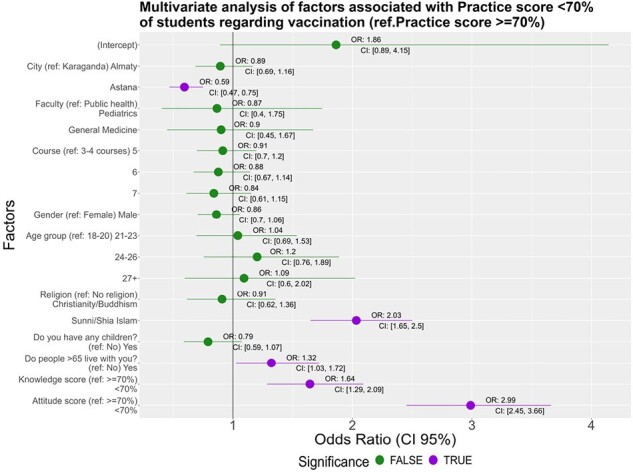
Factors associated with vaccine practice among medical students in Kazakhstan

**Results:**

We interviewed 3,956 students: 1,716 (43%) in Astana, 1,313 (33%) in Almaty, and 927 (24%) in Karaganda. Of these, 74% were female, and 65% were 21-23 years old. Additionally, 89% were studying general medicine, 8% pediatrics, and 3% public health. Fewer than half (38%) reported having received training on vaccination. Adequate KAP scores were 11% for knowledge, 55% for attitudes, and 17% for practices. Most (74%) believed common myths about routine vaccinations, including 62% believing that the flu vaccine causes flu. Knowledge scores < 70% were associated with attitude scores < 70% (OR: 1.72; CI=1.37–2.17) and practice scores < 70% (OR: 1.66; CI=1.30–2.11). Attitude scores < 70% were associated with being male vs female (OR: 1.25; CI=1.07–1.47), living with anyone < 18 years old vs not (OR: 1.37; CI=1.08–1.73), and living in Astana vs Karaganda (OR: 1.69 CI=1.41–2.03). Practice scores < 70% were associated with living with anyone >65 years old vs not (OR: 1.32; CI=1.03–1.72), Islam religion vs no religion (OR: 2.03; CI=1.65–2.50) and having attitude score < 70% vs ≥70% (OR: 2.99; CI=2.45–3.66).

**Conclusion:**

Our study revealed markedly low levels of correct KAP related to routine immunizations. These results demonstrate the need to increase training on vaccination among medical students, especially among pediatric and general medicine students who will be involved in immunizations. Trainings should address common myths about routine immunizations.

**Disclosures:**

**All Authors**: No reported disclosures

